# Next‐generation sequencing of tissue and circulating tumor DNA: Resistance mechanisms to EGFR targeted therapy in a cohort of patients with advanced non‐small cell lung cancer

**DOI:** 10.1002/cam4.3948

**Published:** 2021-06-25

**Authors:** Yujun Zhang, Liwen Xiong, Fangfang Xie, Xiaoxuan Zheng, Ying Li, Lei Zhu, Jiayuan Sun

**Affiliations:** ^1^ Department of Respiratory Endoscopy Shanghai Chest Hospital Shanghai Jiao Tong University Shanghai China; ^2^ Department of Respiratory and Critical Care Medicine Shanghai Chest Hospital Shanghai Jiao Tong University Shanghai China; ^3^ Shanghai Engineering Research Center of Respiratory Endoscopy Shanghai China; ^4^ Department of Pathology Shanghai Chest Hospital Shanghai Jiao Tong University Shanghai China

**Keywords:** epidermal growth factor receptor‐tyrosine kinase inhibitor (EGFR‐TKI), genetic alterations, non‐small cell lung cancer (NSCLC), re‐biopsy

## Abstract

**Background:**

Epidermal growth factor receptor‐tyrosine kinase inhibitor (EGFR‐TKI) has been considered as an effective treatment in epidermal growth factor receptor‐mutant (EGFR‐mutant) advanced non‐small cell lung cancer (NSCLC). However, most patients develop acquired resistance eventually. Here, we compared and analyzed the genetic alterations between tissue assay and circulating tumor DNA (ctDNA) and further explored the resistance mechanisms after EGFR‐TKI treatment.

**Methods and Materials:**

Amplification refractory mutation system‐polymerase chain reaction (ARMS‐PCR), Cobas^®^ ARMS‐PCR and next‐generation sequencing (NGS) were performed on tissue samples after pathological diagnosis. Digital droplet PCR (ddPCR) and NGS were performed on plasma samples. The association between genetic alterations and clinical outcomes was analyzed retrospectively.

**Results:**

Thirty‐seven patients were included. The success rate of re‐biopsy was 91.89% (34/37). The total detection rate of EGFR T790M was 62.16% (23/37) and the consistency between tissue and ctDNA was 78.26% (18/23). Thirty‐four patients were analyzed retrospectively. For tissue re‐biopsy, 24 patients harbored concomitant mutations. Moreover, tissue re‐biopsy at resistance showed 21 patients (21/34, 61.76%) had the concomitant somatic mutation. The three most frequent concomitant mutations were tp53 (18/34, 52.94%), MET (4/34, 11.76%), and PIK3CA (4/34, 11.76%). Meanwhile, 21 patients (21/34, 61.76%) with EGFR T790M mutation. Progression‐free survival (PFS) and overall survival (OS) were better in patients with T790M mutation (*p* = 0.010 and *p* = 0.017) or third‐generation EGFR‐TKI treatment (*p* < 0.0001 and *p* = 0.073). Interestingly, concomitant genetic alterations were significantly associated with a worse prognosis for patients with EGFR T790M mutation receiving third‐generation EGFR‐TKIs (*p* = 0.037).

**Conclusions:**

Multi‐platforms are feasible and highly consistent for re‐biopsy after EGFR‐TKI resistance. Concomitant genetic alterations may be associated with a poor prognosis for patients with EGFR T790M mutation after third‐generation EGFR‐TKIs.

## Graphical abstract

It is feasible to use multi‐platforms to perform detection of EGFR T790M mutation on EGFR‐TKI resistant re‐biopsy tissue and blood samples. The detection consistency of EGFR T790M between tissue and blood samples is high. Concomitant genetic alterations may lead to a worse response to treatment and affect decisions of the sequential therapy strategy.

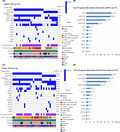

**Abbreviations**
ARMS‐PCR: amplification refractory mutation system‐polymerase chain reaction
CR: complete response
ctDNA: circulating tumor DNA
ddPCR: digital droplet PCR
ECOG: Eastern Cooperative Oncology Group
EGFR: epidermal growth factor receptor
EGFR‐TKI: epidermal growth factor receptor‐tyrosine kinase inhibitor
NGS: next‐generation sequencing
NSCLC: non‐small‐cell lung cancer
ORR: objective response rate
OS: overall survival
PD: progressive disease
PFS: progression‐free survival
PR: partial response
PS: performance status
RECIST: Response Evaluation Criteria in Solid Tumors
ROSE: rapid on‐site evaluation
SD: stable disease
TBB: transbronchial biopsy
TBLB: transbronchial lung biopsy
TBNA: transbronchial needle aspiration
TTNA: transthoracic needle aspiration

## 1 INTRODUCTION

Lung cancer is the leading cause of cancer‐related mortality worldwide.^1^ Among them, non‐small cell lung cancer (NSCLC) accounts for about 85% of lung cancer.^2^ Epithelial growth factor receptor (EGFR) is the most common driver gene in NSCLC, occurring in an estimated 50% of adenocarcinoma cases in Asia. Exon 19 deletion (19del) and exon 21 p.L858R (21L858R) mutation account for about 90% of all EGFR activating mutations and are the most relevant predictors of response to EGFR tyrosine kinase inhibitor (EGFR‐TKI).^4^

However, almost all patients eventually develop acquired drug resistance after EGFR‐TKI treatment. The occurrence of EGFR mutation p.T790M in exon 20 represents the most frequent mechanism of the acquired resistance.^5^ The third‐generation EGFR‐TKI is an irreversible selective TKI, which specifically targets EGFR T790M and EGFR activating mutations and has been proven effective in patients with EGFR T790M‐positive NSCLC following acquired resistance to prior EGFR‐TKIs.^6^, ^7^ This makes re‐biopsy widely accepted in clinical practice.^8^ Through the re‐biopsy, it is possible to effectively understand the cause of drug resistance and provide a basis for follow‐up treatment.

Unfortunately, resistance to third‐generation EGFR‐TKIs also inevitably occurs.^9^, ^10^ Additionally, the detection rate of mutations has increased significantly with the development of next‐generation sequencing (NGS), while the association of concomitant genetic alterations with the treatment response is still uncertain.^11^, ^12^ Therefore, it is essential to explore the re‐biopsy status to identify resistance mechanisms early in patients with targeted drugs.

## 2 MATERIALS AND METHODS

### 2.1 Patient selection

From September 2017 to January 2019, patients with advanced NSCLC who were initially diagnosed with EGFR 19del or EGFR 21L858R mutation were evaluated as progressive disease (PD) after the first‐generation or the second‐generation EGFR‐TKI treatment and were enrolled. The inclusion criteria included (a) 18–80 years old; (b) patients with an initial diagnosis of advanced NSCLC and molecular pathology confirmed the presence of EGFR‐sensitive mutations; (c) clinically accepted first‐ or second‐generation EGFR‐TKI treatment; (d) according to the criteria of solid tumor evaluation criteria (response evaluation criteria in solid tumors, RECIST), clinicians believe that re‐biopsy is necessary to guide the treatment of the patient; (e) patient performance status (PS) rated as score ≤two according to the Eastern Cooperative Oncology Group (ECOG). The exclusion criteria were as follows: (a) the patient received blood transfusion therapy within one month; (b) patients with autoimmune diseases, including but not limited to systemic lupus erythematosus, rheumatoid arthritis, Sjogren's syndrome, etc.; (c) patients with serious diseases were not suitable for biopsy; (d) patients refused to participate in clinical trials; (e) the investigator believed that the patient had other conditions that were not suitable for this clinical trial. The study was approved by the Ethics Committee of Shanghai Chest Hospital. All patients were fully informed and signed informed consent. The clinical trial registration was carried out on ClinicalTrials.gov (NCT03309462).

### 2.2 Sample preparation

Biopsies were performed twice in patients initially diagnosed with advanced NSCLC and in patients with EGFR‐sensitive mutations who had progressed after the first‐ or second‐generation of treatment. Liquid biopsy specimens were collected via standard venipuncture techniques into two tubes (7.5 ml per tube). The circulating tumor DNA (ctDNA) was extracted from the plasma fraction of EDTA blood samples within two hours of the collection as recommended (QIAamp Circulating Nucleic Acid Kit, Qiagen, Hilden, Germany), and gDNA of white cell count (WCC) was extracted using QIAamp DNA Blood mini kit (Qiagen). According to the manufacturer's instructions, the ctDNA was eluted with 120 μL of nuclease‐free water mixed with 3 μL glycogen (20 mg/mL), 1/10 volume of 50 mM triethylamine, precipitated with five volumes of acetone, and reconstituted in 30–50 μL of water. Tissue samples were obtained from small biopsies with multiple techniques, including but not limited to transbronchial biopsy (TBB), transbronchial lung biopsy (TBLB), transbronchial needle aspiration (TBNA), transthoracic needle aspiration (TTNA), and percutaneous lymph node needle biopsy. Then the obtained tissue samples were fixed and embedded. After that, the formalin‐fixed, paraffin‐embedded (FFPE) specimens underwent histological review by hematoxylin‐eosin staining before subjecting to nucleic acid extraction. DNA was purified using a QIAamp DNA FFPE Kit (Qiagen). All DNA quantities were verified using Qubit 3.0 with a dsDNA HS Assay Kit (Life Technologies, Carlsbad, CA, USA).

### 2.3 Gene testing methods

Amplification refractory mutation system‐polymerase chain reaction (ARMS‐PCR) for detecting EGFR mutations was performed via EGFR 21 Mutations Detection Kit (Amoy Diagnostics, Xiamen, China). According to the manufacturer's instructions, DNA was extracted from 10 to 15 unstained FFPE sections, each 5 μm thickness, using QIAamp DNA FFPE tissue kit (Qiagen). The concentration of DNA was measured by SMA4000 spectrophotometer (Merinton, Beijing, China). Cobas^®^ ARMS‐PCR was performed with 10–15 slides of 5 μm paraffin sections subjected to DNA extraction using a Cobas^®^ DNA sample preparation kit (Roche Diagnostics, Indianapolis, IN, USA), and DNA concentration and purity were examined using a Nanodrop UV‐Vis spectrophotometer. DNA integrity was examined by agarose gel electrophoresis. Genetic testing was performed using the human EGFR gene kit Cobas^®^ EGFR Mutation Test v2 (Roche Diagnostics) for EGFR 18–21 exons mutations. For NGS, the library was constructed based on OncoAim™ cancer 223 gene panel (Singlera Genomics, Inc., Shanghai, China) with a total of 1300 exon regions, 456 hotspots, 21 intron regions, and 1 gene promoter. DNA shearing was performed using Covaris M220, followed by end repair, phosphorylation, and adaptor ligation. Fragments of size 200–400 bp were selected using Agencourt AMPure beads (Beckman Coulter, Brea, CA, USA) followed by hybridization with capture probes baits, hybrid selection with magnetic beads and PCR amplification. A bioanalyzer high‐sensitivity DNA assay was performed to assess the quality and size of the fragments. Fifty ng of DNA was used for library construction. Twelve PCR cycles were used for library amplification. The indexed samples were sequenced on Hiseq500 sequencer (Illumina, Inc., San Diego, CA, USA) with paired reads (read length 150 bp). The sequencing data in the FASTQ format were mapped to the human genome (hg19) using BWA aligner 0.7.10. Local alignment optimization, variant calling and annotation were performed using GATK 3.2, MuTect, and VarScan, respectively. DNA translocation analysis was performed using both Tophat2 and Factera 1.4.3. Gene‐level copy number variation was assessed using a *t* statistic after normalizing reads depth at each region by total reads number and region size, and correcting GC‐bias using a LOESS algorithm.

### 2.4 Follow‐up

Clinical follow‐up assessments including physical examinations, imaging, and routine laboratory tests were performed every 4 weeks. Tumor response was assessed according to the RECIST (version) 1.1 by experienced investigators and categorized as complete response (CR), partial response (PR), stable disease (SD) or PD. Progression‐free survival (PFS) was defined as the duration between the initiation of EGFR‐TKIs and progression of disease or death for any cause, whichever occurred first. Similarly, overall survival (OS) was calculated from the date of starting EGFR‐TKIs to the date of death for any cause or last follow‐up (censored patient). Patients who were still alive were censored on their date of the last follow‐up per chart review. The cut‐off date for analysis was 25 April 2019.

### 2.5 Statistical analysis

Frequency, percentage, average ±SD, median (range) were presented as appropriate. Survival was estimated with Kaplan–Meier methodology and compared with the use of log‐rank test between different groups. *P* values were calculated using Fisher's exact test or Pearson test for categorical variables or continuous variables, respectively. Wilcoxon test was used for comparing continuous variables to binary variables. Statistical analysis was performed using SPSS v25 (IBM Corporation, NY, USA). A *P* value less than 0.05 was considered statistically significant.

## 3 RESULTS

### 3.1 Patients’ characteristics

**FIGURE 1:**
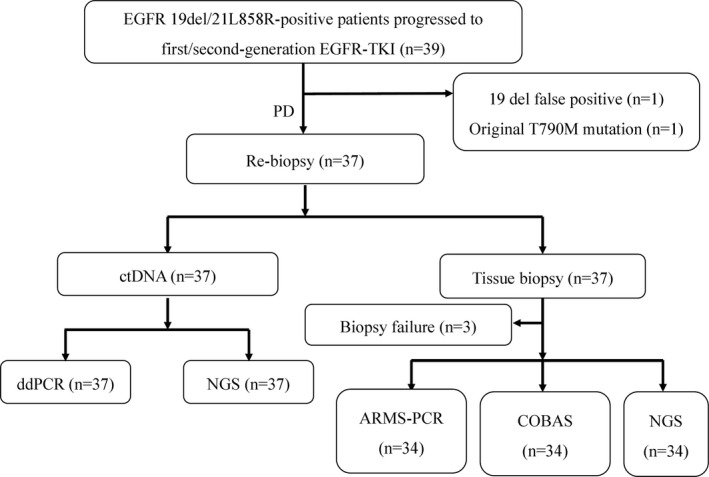
Flow chart of eligible population. A total of 39 patients with lung cancer diagnosed with EGFR 19del or EGFR 21L858R mutation positive were resistant to the first‐ or second‐generation EGFR‐TKIs after treatment and enrolled. Among them, when NGS re‐examined the genes in the first biopsy sample, 1 case was excluded due to the result indicating that EGFR 19del was false positive, and 1 case was excluded due to the result indicating mutation containing EGFR 20T790M. Thirty‐seven patients had tissue gene testing and blood gene testing. ARMS‐PCR, amplification refractory mutation system‐polymerase chain reaction; ctDNA, circulating tumor DNA; ddPCR, digtal droplet polymerase chain reaction; EGFR, epidermal growth factor receptor; EGFR‐TKI, epidermal growth factor receptor‐tyrosine kinase inhibitor; NGS, next‐generation sequencing; PD, progressive disease.

In this study, 39 patients were enrolled and 34 patients were included in the retrospective analysis. The enrollment process was shown in Figure 1. Two patients were excluded, due to one 19del false positive and one T790M mutation at baseline. Thirty‐seven patients were successfully tested for plasma with a success rate of 100% (37/37), while three patients failed to perform tissue re‐biopsy due to few tumor cells by pathological examination. The characteristics of initial and secondary biopsy of enrolled patients were summarized in Tables 1 and 2. Ultimately, all enrolled patients received the first‐generation TKI treatment only. Thirty‐four patients’ clinical characteristics for retrospective analysis were shown in Table 3. Pathology revealed only one adenosquamous cell carcinoma. The others were adenocarcinoma (97.06%, 33/34). The common EGFR activating alterations were largely represented (67.65%, 23/34, 19del; 32.35%, 11/34, 21L858R). During first‐line treatment, median PFS resulted 13 months. After progression, 70.59% (24/34) of patients received third‐generation EGFR‐TKI treatment, and median PFS resulted 6 months. The first patient started third‐generation EGFR‐TKI treatment on 24 October 2017 and the last one assumed the first dose on 17 August 2018. The data cut‐off for this analysis was 25 April 2019.

**TABLE 1:** Initial biopsy baseline information (n = 39).

Characteristics	Number
Gender, n (%)	
Male	21 (53.85%)
Female	18 (46.15%)
Age, median (range), years	61 (38–79)
Smoking history, n (%)	
Yes	12 (30.77%)
No	27 (69.23%)
Pathology, n (%)	
Adenocarcinoma	38 (97.44%)
Adenosquamous carcinoma	1 (2.56%)
Molecular pathology, n (%)	
EGFR 19del	26 (66.67%)
EGFR 21L858R	13 (33.33%)
Treatment, n (%)	
First‐generation EGFR‐TKI	36 (92.31%)
Chemotherapy +first‐generation EGFR‐TKI	3 (7.69%)
PFS^a^, median (range), months	13.0 (1–35)
Sites, n (%)	
Lung lesions	26 (66.67%)
Intrathoracic metastatic lymph nodes	3 (7.69%)
Extrathoracic metastatic lymph nodes	6 (15.38%)
Pleural effusion	4 (10.26%)
Methods of initial biopsy, n (%)	
TBB	10 (25.64%)
EBUS‐TBNA	3 (7.69%)
TBLB	1 (2.56%)
TTNA	10 (25.64%)
Percutaneous needle aspiration of lymph nodes	6 (15.38%)
Surgery	5 (12.82%)
Pleural effusion	4 (10.26%)

Abbreviations: EBUS‐TBNA, endobronchial ultrasound transbronchial needle aspiration; EGFR, epidermal growth factor receptor; EGFR‐TKI, epidermal growth factor receptor‐tyrosine kinase inhibitor; PFS, progression‐free survival; TBB, transbronchial biopsy; TBLB, transbronchial lung biopsy; TTNA, transthoracic needle aspiration.

^a^ PFS was determined from the starting date of the first‐generation EGFR‐TKI treatment to the date of disease progression.

**TABLE 2:** Re‐biopsy baseline information (n = 37).

Characteristics	Number
Pathology, n (%)	
Adenocarcinoma	30 (81.08%)
Squamous cell lung cancer	3 (8.11%)
Small cell lung cancer	1 (2.7%)
Inadequate tumor cells	3 (8.11%)
Clinical staging, n (%)	
IIIA	3 (8.11%)
IIIB	2 (5.41%)
IV	32 (86.49%)
Metastasis, n (%)	
Bone metastasis	11 (29.73%)
Brain metastasis	16 (43.24%)
Pleural metastasis	8 (21.62%)
Other distant metastases	18 (48.65%)
Treatment, n (%)	
Third‐generation EGFR‐TKI	27 (72.97%)
Radio/chemotherapy	10 (27.03%)
PFS^a^, median (range), months	6 (0–23)
OS, median (range), months	13 (0–23)
Sites, n (%)	
Lung lesions	23 (62.16%)
Intrathoracic metastatic lymph nodes	8 (21.62%)
Extrathoracic metastatic lymph nodes	6 (16.22%)
Methods of re‐biopsy, n (%)	
TBB	9 (24.32%)
EBUS‐TBNA	8 (21.62%)
TBLB	6 (16.22%)
TTNA	8 (21.62%)
Percutaneous needle aspiration of lymph nodes	6 (16.22%)

Abbreviations: EBUS‐TBNA, endobronchial ultrasound transbronchial needle aspiration; EGFR‐TKI, epidermal growth factor receptor‐tyrosine kinase inhibitor; OS, overall survival; PFS, progression‐free survival; TBB, transbronchial biopsy; TBLB, transbronchial lung biopsy; TTNA, transthoracic needle aspiration.

^a^ PFS was determined from the starting date of the third‐generation EGFR‐TKI treatment and/or other treatments to the date of disease progression, or the last follow‐up time for those who have not reached disease progression.

**TABLE 3:** Patients’ clinical characteristics for retrospective analysis.

Characteristic	NO. (%)
Age, mean ±SD, years	58.5 ± 9.31
Sex, n (%)	
Male	17 (50%)
Female	17 (50%)
Smoking history, n (%)	
Never smoker	27 (79.41%)
Smoker	7 (20.59%)
Clinical stageing for retrospective analysis, n (%)	
Ⅲ	5 (14.71%)
Ⅳ	29 (85.29%)
T stage, n (%)	
T1	1 (2.94%)
T2	22 (64.71%)
T3	2 (5.88%)
T4	9 (26.47%)
Lymph node metastasis, n (%)	
N0	1 (2.94%)
N2	16 (47.06%)
N3	17 (50%)
Distant metastasis, n (%)	
No	5 (14.71%)
Yes	29 (85.29%)
Metastatic sites, n (%)	
Brain	10 (29.41%)
Bone	17 (50%)
Liver	2 (5.88%)
Adrenal	4 (11.76%)
Pericardium	2 (5.88%)
ECOG PS, n (%)	
0	12 (35.29%)
1	22 (64.71%)
Baseline pathological classification, n (%)	
Adenocarcinoma	33 (97.06%)
Adenosquamous carcinoma	1 (2.94%)
Re‐biopsy pathological classification, n (%)	
Adenocarcinoma	30 (88.24%)
Squamous carcinoma	3 (8.82%)
Small cell lung cancer	1 (2.94%)
EGFR mutation, n (%)	
19del	23 (67.65%)
L858R	11 (32.35%)
First line EGFR‐TKI, n (%)	
gefitinib	8 (23.53%)
erlotinib	3 (8.82%)
icotinib	21 (61.76%)
others	2 (5.88%)
Post‐TKI treatment, n (%)	
Third‐generation EGFR‐TKI	24 (70.59%)
Radio/chemotherapy	10 (29.41%)

Abbreviations: ECOG PS: Eastern Cooperative Oncology Group performance status; EGFR, epidermal growth factor receptor; EGFR‐TKI, epidermal growth factor receptor‐tyrosine kinase inhibitor.

### 3.2 Genetic alterations in ctDNA and tissue of enrolled patients

**FIGURE 2:**
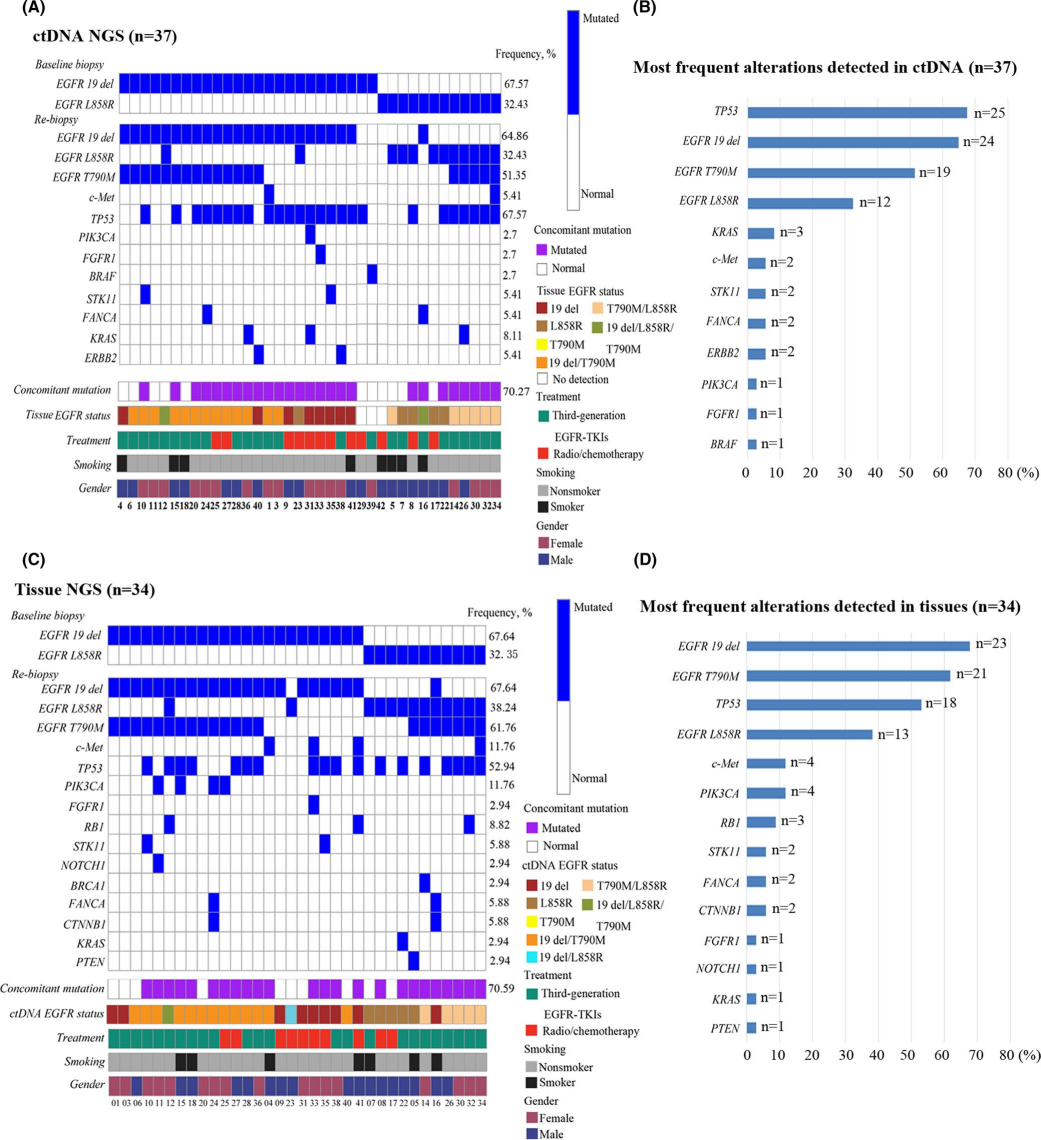
Genetic alterations of enrolled patients by ctDNA and tissue NGS. (A) Oncoprint of ctDNA NGS alterations (n = 37). Synonymous alterations and variants of unknown significance were excluded. All 37 patients were tested for ctDNA, but only 34 of them were also tested for tissue NGS. Each vertical bar represents a patient. (B) Most frequent alterations identified by plasma‐derived ctDNA NGS (n = 37). (C) Oncoprint of tissue NGS (n = 34). (D) Most frequent alterations identified by tissue NGS (n = 34). ctDNA, circulating tumor DNA; EGFR, epidermal growth factor receptor; EGFR‐TKI, epidermal growth factor receptor‐tyrosine kinase inhibitor; NGS, next‐generation sequencing.

Of the 37 patients who had plasma sent for ctDNA NGS, 34 (91.89%) also had tissue sent for solid tumor NGS. Out of 34 patients with samples sent for both ctDNA and tissue NGS, 34 had shared at least 1 alteration identified by both tissue NGS and ctDNA analysis. In Figure 2A, the most frequent alterations detected by ctDNA NGS were displayed. The three most frequent ctDNA alterations involved the following genes: TP53 (67.57%, 25/37), followed by KRAS (8.11%, 3/37) and amplification of c‐Met (5.41%, 2/37) (Figure 2B). The most frequent alterations detected by tissue NGS involved the following genes: TP53 (52.94%, 18/34), amplification of c‐Met (11.76%, 4/34) and PIK3CA (11.76%, 4/34) (Figure 2C and 2D). In total, 83.78% (31/37) of patients harbored concomitant mutations, 70.27% (26/37) by ctDNA and 70.59% (24/34) by tissue NGS. Patients with a history of smoking (85.71% [6/7] vs 66.67% [18/27]) were found in tissue with a higher incidence of concomitant mutations, but not in ctDNA (37.5% [3/8] vs 79.31% [23/29]).

**FIGURE 3:**
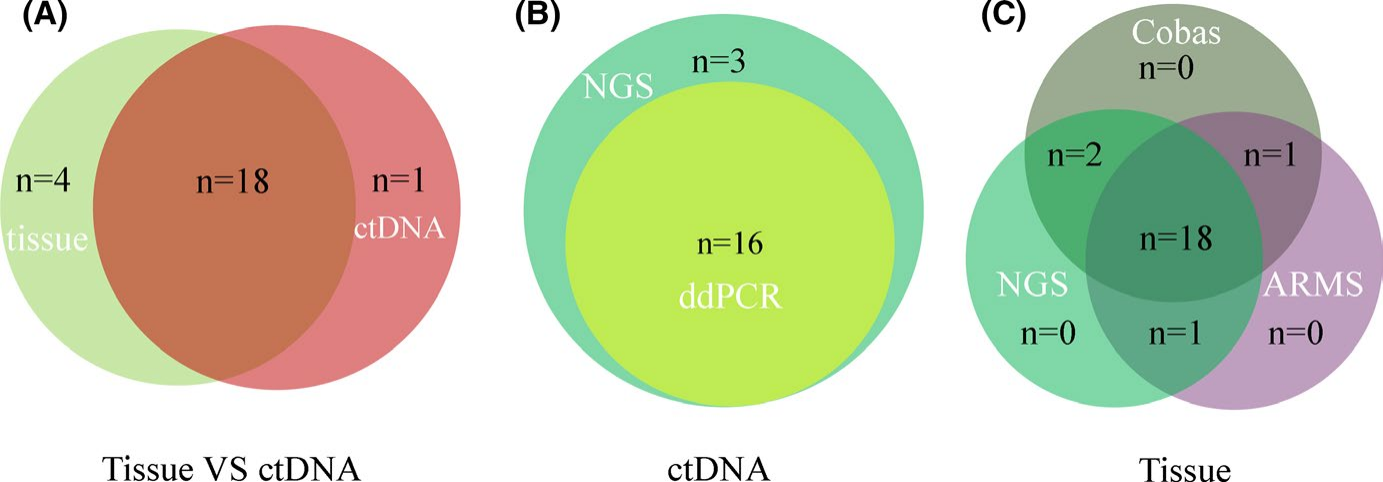
The detection efficiency of different detection platforms for EGFR T790M mutation. (A) EGFR T790M mutation was detected and compared by both ctDNA and tissue assays and as illustrated by the Venn diagrams. (B) EGFR T790M mutation was detected and compared by NGS and ddPCR in plasma samples. (C) EGFR T790M mutation was detected and compared by ARMS‐PCR, Cobas^®^ ARMS‐PCR and NGS in tissue samples. ARMS‐PCR, amplification refractory mutation system‐polymerase chain reaction; ctDNA, circulating tumor DNA; ddPCR, digtal droplet polymerase chain reaction; EGFR, epidermal growth factor receptor; NGS, next‐generation sequencing.

A total of 23 cases of EGFR T790M mutation in plasmas and tissues were detected after first‐line EGFR‐TKI treatment. The total positive rate was 62.16% (23/37). The positive rate of tissue samples to detect EGFR T790M mutation was 64.71% (22/34), and the positive rate of plasma samples to detect EGFR T790M mutation was 51.35% (19/37). One of the patients had a positive mutation in plasma, while the tissue was negative. This patient was treated with Osimertinib in the follow‐up treatment. The best efficacy was evaluated as PR and PFS was 7.0 months. Therefore, we combined the efficacy and test results to consider the tissue test results as false negative, suggesting that tissue and ctDNA assay provided complementary results. The remaining 18 patients with positive blood tests were consistent with those having positive tissue tests (Figure 3A). Therefore, the consistency rate between tissue and plasma for EGFR T790M mutation was 78.26% (18/23). Tissue and plasma also showed a high consistency for some other frequently mutated genes, such as TP53, PIK3CA, c‐Met, STK11, FANCA, and KRAS. (Figure S1). In addition, EGFR T790M mutation in plasma samples were tested and compared with ddPCR and NGS as illustrated by the Venn diagrams. Tissues were detected and compared by ARMS‐PCR, Cobas^®^ ARMS‐PCR, and NGS (Figure 3B and 3C). The consistency rate in plasma and tissue for different platforms were 84.21% (16/19) and 78.26% (18/23), respectively.

### 3.3 Treatment outcome

**FIGURE 4:**
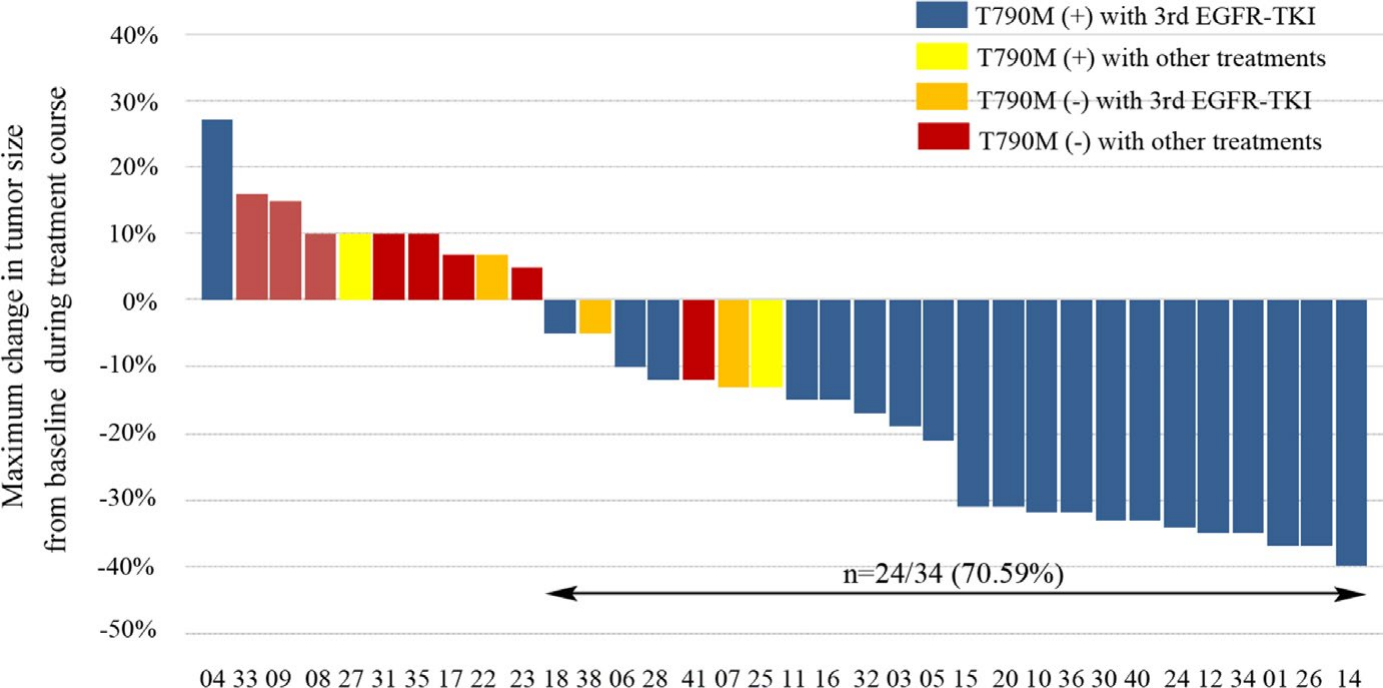
Maximum change in tumor size from baseline in individual patients over the course of treatment. Changes in tumor size (diameter) were assessed in patients with or without EGFR T790M mutation treated with the third‐generation EGFR‐TKIs or other treatments. Tumor shrinkage relative to baseline was observed in 70.59% of patients. EGFR‐TKI, epidermal growth factor receptor‐tyrosine kinase inhibitor.

**FIGURE 5:**
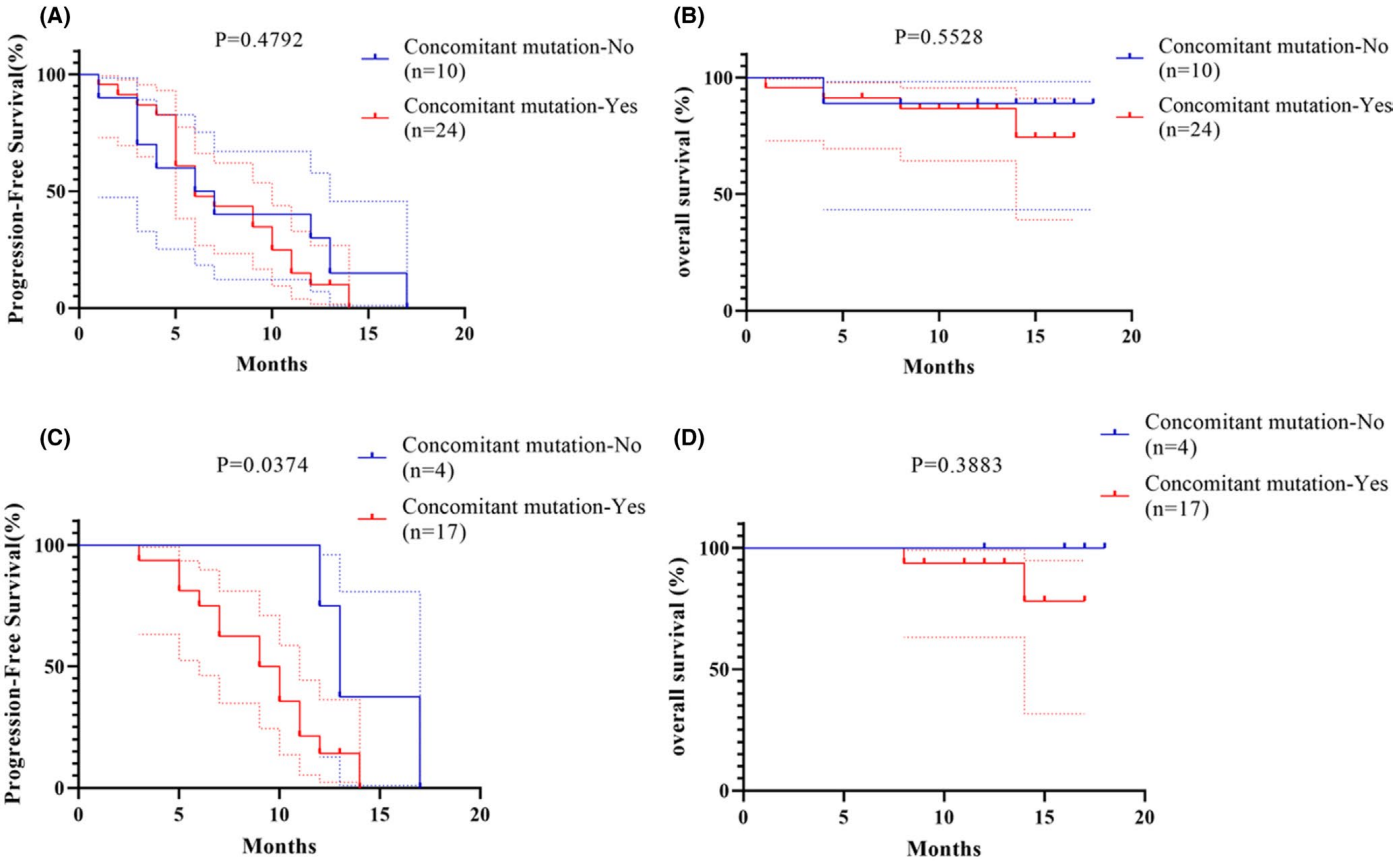
Survivals analysis of patients after resistance to first‐generation TKI therapy. (A) Kaplan‐Meier curves of PFS in 34 patients with first‐generation EGFR‐TKI treatment whose tissue re‐biopsy had concomitant mutations compared with those without concomitant mutations. (B) Kaplan‐Meier curves of OS in 34 patients with first‐generation EGFR‐TKI treatment whose tissue re‐biopsy had concomitant mutations compared with those without concomitant mutations. (C) Kaplan‐Meier curves of PFS in 21 patients with EGFR T790M mutation whose tissue re‐biopsy had concomitant mutations compared with those without concomitant mutations after receiving third‐generation EGFR‐TKIs treatment. (D) Kaplan‐Meier curves of OS in 21 patients with EGFR T790M mutation whose tissue re‐biopsy had concomitant mutations compared with those without concomitant mutations after receiving third‐generation EGFR‐TKIs treatment. EGFR, epidermal growth factor receptor; EGFR‐TKI, epidermal growth factor receptor‐tyrosine kinase inhibitor; OS, overall survival; PFS, progression‐free survival.

Of the 34 patients in the retrospective analysis, 24 received third‐generation EGFR‐TKI treatment and 10 received others after progression with first‐line EGFR‐TKI treatment. Twenty‐four (70.59%, 24/34) of patients exhibited prominent tumor shrinkage during treatment. Among them, 22 received third‐generation EGFR‐TKI treatment, revealing the efficacy of third‐generation EGFR‐TKIs for patients with resistance to first‐line treatment (Figure 4). Thirteen partial responses were observed and all occurred in EGFR T790M‐positive patients (contained one who was detected only by ctDNA assay and one was not detected by the tissue NGS but was detected on other platforms), the objective response rate (ORR) was 54.17% as shown in Table 4 (*p* = 0.003). Comparing with third‐generation EGFR‐TKI treatment, radio/chemotherapy (others) significantly showed shorter PFS (*p* < 0.001, median survival, 4.0 months, ratio, 0.25[95% CI, 0.19–0.85 months] vs 10.0 months, ratio, 4[95%CI, 1.17–5.34]; HR, 4.49[95%CI, 1.36–14.76]) and OS (*p* = 0.058, HR, 4.72[95%CI, 0.61–36.57]). Similarly, T790M‐negative patients significantly showed shorter PFS than patients with T790M mutation (*p* = 0.010, median survival, 5.0 months, ratio, 0.5[95% CI, 0.23–1.08] vs 10.0 months, ratio, 2.5[95%CI, 0.92–4.33]; HR, 2.37[95%CI, 0.92–6.09]), while OS was not statistically significant (Table 4). There was a significant difference in the response rate between the EGFR T790M‐positive and EGFR T790M‐negative patients (57.14% vs 7.69%, respectively; *p* = 0.004, chi‐squared test). Furthermore, concomitant genetic alterations were significantly associated with a poor PFS comparing with those without concomitant genetic alterations for patients receiving third‐generation EGFR‐TKIs with EGFR T790M mutation as shown in Figure 5C (*p* = 0.0374, median survival, 13.0 months, ratio,1.37 [95% CI, 0.39–4.76] vs 9.5 months, ratio, 0.73[95%CI, 0.21–2.54]; HR, 0.33 [95%CI, 0.12–0.87]).

**TABLE 4:** Treatment outcomes for patients with EGFR‐mutant advanced non‐small cell lung cancer treated with EGFR‐TKIs.

Variable	NO. (%)	Objective Response		Progression‐Free Survival				Overall Survival			
				Univariate		Multivariable^a^		Univariate		Multivariable^a^	
		NO. (%)	P Value	HR (95% CI)	P Value	HR (95% CI)	P Value	HR (95% CI)	P Value	HR (95% CI)	P Value
Post‐TKI treatments											
Third‐generation EGFR‐TKIs	24 (70.59%)	13 (54.17%)	0.003	1 (Reference)	<0.0001	/	/	1 (Reference)	0.058	/	/
Radio/Chemotherapy	10 (29.41%)	0 (0.0%)		4.49 (1.36–14.76)		/		4.72 (0.61–36.57)		/	
EGFR status^b^											
Exon 19 deletion	23 (67.65%)	9 (39.13%)	0.877	1 (Reference)	0.272	1(Reference)	0.811	1 (Reference)	0.874	1 (Reference)	0.747
Exon 21 mutation	11 (32.35%)	4 (36.36%)		1.46 (0.66–3.23)		0.89(0.34–2.30)		1.15 (0.19–7.12)		0.75 (0.13–4.38)	
EGFR T790M mutation^b^											
Positive	21 (61.76%)	12 (57.14%)	0.004	1 (Reference)	0.010	1(Reference)	0.000	1 (Reference)	0.584	1 (Reference)	0.437
Negative	13 (38.24%)	1 (7.69%)		2.37 (0.92–6.09)		9.92(3.13–31.49)		0.44 (0.07–2.72)		1.97 (0.36–10.89)	
Concomitant mutation^b^											
Yes	24 (70.59%)	9 (37.5%)	0.891	1 (Reference)	0.479	1 (Reference)	0.174	1 (Reference)	0.553	1 (Reference)	0.836
No	10 (29.41%)	4 (40%)		0.78 (0.37–1.64)		0.53 (0.21–1.33)		0.53 (0.08–3.44)		0.82 (0.13–5.29)	

Abbreviations: EGFR, epidermal growth factor receptor; TKI, tyrosine kinase inhibitor.

^a^ Age (stratified by 65 years old), sex, history of smoking, tumor grade and stage, EGFR status, EGFR T790M mutation, and concomitant mutations were entered into the multivariable Cox proportional hazards regression model.

^b^ Based on tissue NGS results.

### 3.4 Concomitant alteration status with third‐generation EGFR‐TKI treatment

All patients progressed after the initial TKI treatment, and 24 of them were subsequently treated with third‐generation EGFR‐TKI (Table 5). Analysis of the putative mechanism of initial resistance in these patients showed that 21 patients with EGFR T790M positive mutations with known activation of bypass signaling pathways were observed. Three with PIK3CA mutation, two with MET amplification, one with PTEN deletion, and one with STK11 mutation were included, excepting one with KRAS activating mutation in EGFR T790M‐negative patients. The other concomitant genetic alterations, including TP53, RB1, NOTCH1, FANCA, CTNNB1, and BRCA1, were also detected. After third‐generation EGFR‐TKI treatment, 22 patients presented with PD and two patients presented with SD. Unfortunately, we did not perform a third biopsy on patients who were assessed as PD after treatment with third‐generation EGFR‐TKIs. It is possible that the original resistance mechanism, such as the activation of some known alternative signaling pathways, has an impact on the resistance of the third‐generation TKI. However, for patients whose initial resistance mechanism is unknown (#7) and patients who only show EGFR T790M mutations (#1, #3, #6, #40), a third biopsy may be necessary.

**TABLE 5:** Tissue re‐biopsy results after progression with initial TKI treatment and the response to subsequent third‐generation EGFR‐TKIs.

T790M+					T790M‐				
patients	Mutations	Best Response	progression	PFS	patients	Mutations	Best Response	progression	PFS
#1	n.d	PR	yes	17	#7	n.d	SD	yes	3
#3	n.d	PR	yes	12	#22	TP53, KRAS	SD	yes	6
#4	c‐MET	PD	yes	0	#38	TP53	SD	yes	6
#5	PTEN	SD	yes	3					
#6	n.d	SD	yes	13					
#10	TP53, STK11	PR	yes	9					
#11	PIK3CA, NOTCH1	SD	yes	11					
#12	TP53, RB1	PR	yes	14					
#14	BRCA1	PR	yes	10					
#15	PIK3CA, TP53	PR	yes	5					
#16	FANCA, CTNNB1	SD	yes	12					
#18	TP53	SD	yes	13					
#20	TP53	PR	no	12					
#24	PIK3CA, FANCA, CTNNB1	PR	yes	7					
#26	TP53	PR	yes	11					
#28	TP53	SD	yes	9					
#30	TP53	PR	yes	10					
#32	TP53, RB1	SD	yes	5					
#34	c‐MET, TP53	PR	yes	6					
#36	TP53	PR	no	9					
#40	n.d	PR	yes	7					

Abbreviations: EGFR, epidermal growth factor receptor; n.d, no detection; PD, progressive disease; PFS, progression‐free survival; PR, partial response; SD, stable disease.

## 4 DISCUSSION

Epidermal growth factor receptor mutation has long been considered as the most important prognostic factor in patients with advanced NSCLC in the EGFR‐TKI era. EGFR‐TKIs were more effective in treating advanced NSCLC than chemotherapy. However, as resistance develops, re‐biopsy will play a significant role in guiding the use of third‐generation EGFR‐TKIs. Also, it provides a better understanding of the underlying resistance mechanisms for third‐generation EGFR‐TKIs to optimize clinical outcomes. Therefore, we prospectively enrolled 39 patients to study the status of re‐biopsy and retrospectively analyzed 34 EGFR‐mutant patients, who were diagnosed with advanced NSCLC after failure to first‐line EGFR‐TKI treatment.

A total of 34 pairs of tissue‐to‐blood paired specimens were successfully biopsied, and histopathological typing and gene detection of tissue, and blood were performed with a success rate of 91.89% (34/37). The success rate reported in previous biopsy studies ranged from 73% to 95%, and our results were similar to previous studies.^13^, ^14^, ^15^, ^16^, ^17^ The reason for our higher success rate may be that we performed more than three biopsy times per lesion. For the three cases that failed to obtain enough tumor tissues for gene detection, the puncture sites were all primary lung lesions, and the pathological characteristics suggested that blood clots and some scattered lung tissues were presented under the microscope, but no tumor cells were observed. For biopsy, the number and proportion of tumor cells cannot be guaranteed, even though some heterotypic cells may exist in the on‐site cytology. This also indicates the difficulty of re‐biopsy after the first‐generation EFGR‐TKI resistance. Another reason for the lack of tumor cells in the re‐biopsy samples may be due to the heterogeneity of the tumor tissue. Previous studies have shown that larger differences can occur between primary tumors and their metastases or tumors of different pathological subtypes of the same tissue.^18^ Xie et al. proved that there was no significant difference in PFS between patients with partially matched mutational profiles between primary tumors and metastatic lymph nodes and those having 100% concordance rate. It indicated that the genetic profiles of both primary lesions and metastatic lymph nodes could be a guidance of NSCLC‐targeted therapy. As well as other studies, it was confirmed that the genetic mutations in the primary lung and metastases were similar in patients with advanced lung cancer.^19^, ^20^, ^21^ Unfortunately, we were unable to confirm another re‐biopsy in these three patients, so we were not sure whether we could obtain adequate, effective and qualified tissue specimens for histopathological typing and genetic testing. However, it may be considered that if the primary lung lesion biopsy fails to obtain a qualified specimen, other metastatic lesions and metastatic lymph nodes may be considered for genetic testing to guide follow‐up treatment.

In this study, we detected and identified potentially actionable alterations using both blood‐derived ctDNA and tumor tissue by different platforms, especially for EGFR T790M mutation. Compared with traditional detection methods, NGS technology has better detection efficiency and higher throughput through tissue and blood samples. It can detect mutations, insertions, rearrangements and copy number variations quantitatively at the same time, and also significantly save cost and time in large‐scale sequencing. Concordance rates between tissue and ctDNA NGS appeared high here, although there were differences in genomic alterations detected. However, compared with the efficacy of genetic testing for tissue biopsy specimens, the efficacy of blood test for EGFR T790M still has more false negatives, resulting in lower sensitivity. Our previous studies have found that subclones with different mutations have different DNA releasing abilities, suggesting that this may be one of the reasons for the differences in mutations between tumor biopsy samples and blood samples.^22^ Meanwhile, there was also one patient with EGFR T790M mutation in plasma but negative in tissue. We combined the efficacy with the test results to consider that the tissue test results were also false negative, suggesting that tissue and ctDNA assay provided complementary results.^23^ Therefore, National Comprehensive Cancer Network guideline recommend that when the patient's physical condition is tolerable and tissue specimens are available, tissue biopsy should be preferred as the first choice for re‐biopsy after the EGFR‐TKI resistance.^8^ When tissue specimens are not available or tissue specimens are unqualified and patients refuse to perform, in the case of a re‐biopsy, liquid biopsy techniques can be a powerful complement.

For genetic alterations and resistance mechanisms, we retrospectively analyzed 34 patients with mutations detected by both ctDNA and tissue NGS. Among them, 88.23% (30/34) of patients harbored concomitant mutations detected by at least one NGS assay. The co‐occurring genetic alterations were negatively correlated with the response to the treatment, which may be plausibly due to the bypass activation of survival signaling pathways or tumor heterogeneity. Consistent with recent studies,^12^ patients with 19del survived longer than patients with 21L858R mutation. Of interest, a higher incidence of concomitant mutations in patients was also detected with 21L858R, although, there was no significant difference which was not shown in the results. As we know, the presence of EGFR T790M mutation in resistant patients after first‐line EGFR‐TKI therapy was significantly associated with better efficacy of third‐generation EGFR‐TKIs, which provides a rationale for the superiority of third‐generation EGFR‐TKI therapy over other therapies. In multivariable analysis, the EGFR T790M mutation was still significantly associated with survival.

To study the potential mechanisms of resistance, re‐biopsy at progression to third‐generation EGFR‐TKIs was performed in 24 patients. Activation of known bypass signaling pathways as mechanism of resistance was represented, and one patient with STK11 mutation, one with PTEN deletion, one with KRAS mutation, two with MET amplification and three with PIK3CA mutation were observed. STK11 mutation presented accompanying with TP53 mutation in an EGFR T790M‐positive patient, which had been reported as a mediator of the cold tumor immune microenvironment and a major driver of primary resistance to PD‐1 axis inhibitors in nonsquamous lung adenocarcinoma.^24^ PTEN loss was previously described as a mechanism of resistance to first‐generation EGFR‐TKIs.^25^ While Kim et al. reported a following increase of the proportion of tumors with PTEN deletions in post‐treatment tumors and the gradual increase of PTEN deletions might contribute to focal progression to Osimertinib.^26^ The MET amplification was already mentioned as a possible mechanism of resistance to osimertinib, which has been considered as the very common findings of acquired resistance under first‐generation EGFR‐TKIs,^27^, ^28^, ^29^ and described in the literature at frequencies ranging from 5% to 50%.^30^, ^31^ Particularly, activating mutations of the catalytic subunit alpha (PIK3CA) of PI3 K lipid kinases family was associated with poor PFS in our cohort including a case of small‐cell lung cancer (SCLC) transformation, since activation of PI3 K/AKT/mTOR signaling pathway was not mutually exclusive with other carcinogenic driving mechanisms. The shorter median survival time in patients with co‐existing PIK3CA and EGFR mutations suggested a possible synergistic effect due to stronger activation of relevant downstream signals.^32^, ^33^ The results suggested a potential role of PIK3CA‐inhibitor, alone or in combination, to accurately overcome this resistance.

The main limitations of this study were sample size, single center design, and lack of continuous biopsy. Furthermore, data from our NGS hotspots (excluding other mutations, copy number changes, or chromosomal abnormalities) may represent an underestimation of concomitant mutations and impede further analysis of signaling pathways or cloning.

## 5 CONCLUSIONS

In conclusion, using multi‐platforms to perform detection of EGFR T790M mutation on EGFR‐TKI resistant re‐biopsy tissue and blood samples is feasible, and the consistency between tissue and blood samples is high. They can provide complementary results mutually. Our study also highlights the importance of re‐biopsy and molecular diagnosis during disease progression in patients treated by EGFR‐TKIs for oncogene‐addicted NSCLC. The concomitant genetic alterations may affect response to treatment and decision of sequential therapy strategy.

## CONFLICT OF INTEREST

The authors declare that they have no competing interests.

## ETHICS APPROVAL AND CONSENT TO PARTICIPATE

The study was approved by the Ethics Committee of Shanghai Chest Hospital, and the ethical approval number was KS1703. All patients were fully informed and signed informed consent. The clinical trial regitration was carried out on ClinicalTrials.gov. (NCT03309462).

## Supporting information

Fig S1

## Data Availability

The datasets used and/or analyzed during the current study are available from the corresponding author on reasonable request.
