# Supplementary figures and images for: Next‐generation sequencing of tissue and circulating tumor DNA: Resistance mechanisms to EGFR targeted therapy in a cohort of patients with advanced non‐small cell lung cancer
Source: Cancer Med. 2021 Jun 25;10(14):4697–709. doi: 10.1002/cam4.3948 (PMC8290257; doi:10.1002/cam4.3948)

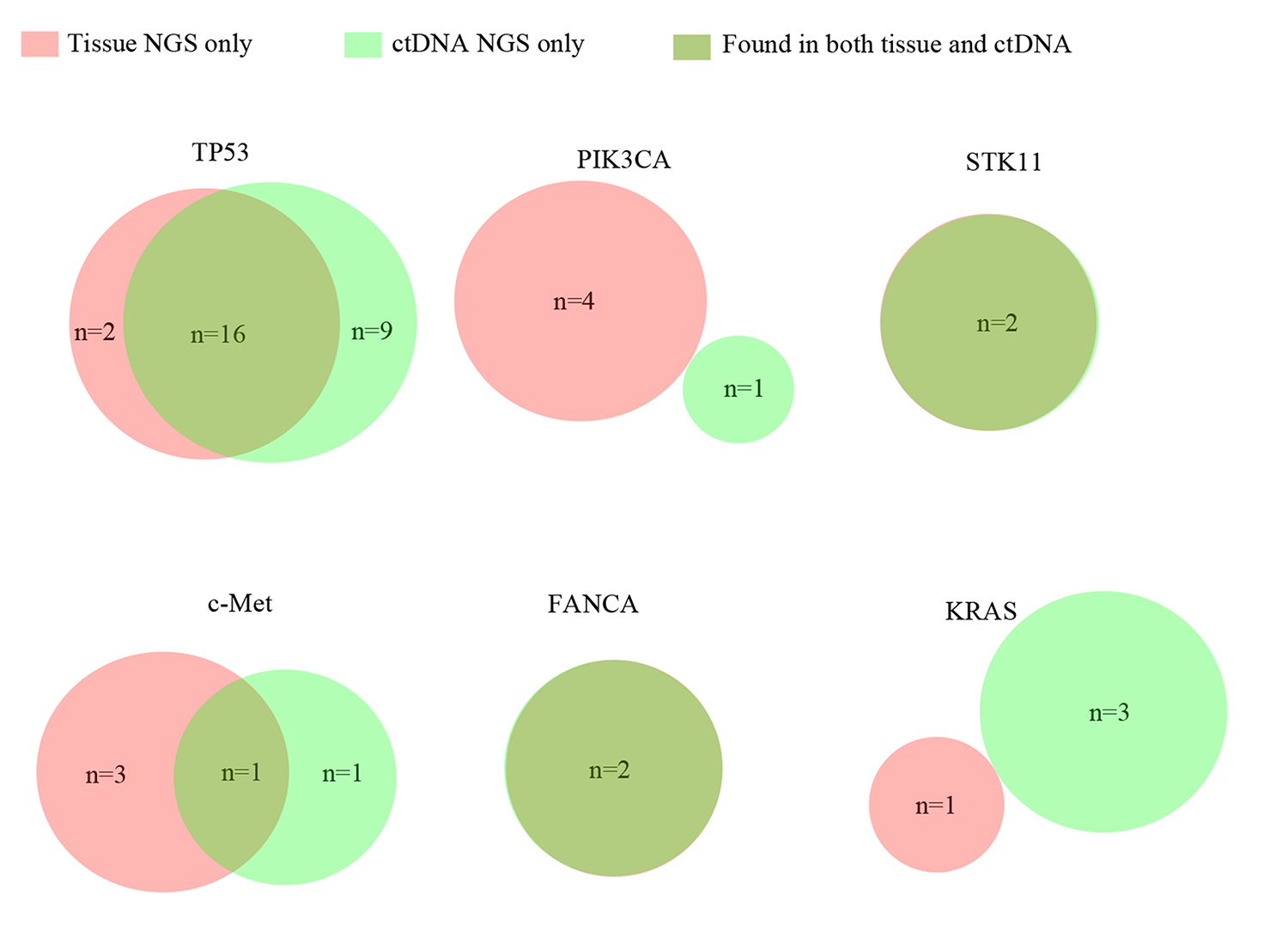

Supplement: Supplementary file 1 — Fig S1 [file CAM4-10-4697-s001.tif]
